# Factors affecting length of stay according to bronchopulmonary dysplasia severity: a nationwide cohort study in Korea

**DOI:** 10.1007/s12519-023-00794-8

**Published:** 2024-02-15

**Authors:** Hye Mi Lee, Jeongmin Shin, Sae Yun Kim, So Young Kim

**Affiliations:** 1 Department of Pediatrics, College of Medicine, The Catholic University of Korea, Seoul, Republic of Korea; 2 Present Address: Department of Pediatrics, Yeouido St. Mary’s Hospital, College of Medicine, The Catholic University of Korea, 10, 63-ro, Yeongdeungpo-gu, Seoul, 07345 Republic of Korea

**Keywords:** Bronchopulmonary dysplasia, Length of stay, Very low birth weight

## Abstract

**Background:**

Longer hospitalizations for preterm infants with bronchopulmonary dysplasia (BPD) delay developmental outcomes, increase the risk for hospital-acquired complications, and exert a substantial socioeconomic burden. This study aimed to identify factors associated with an extended length of stay (LOS) at different levels of severity of BPD.

**Methods:**

A cohort study was conducted using the Korean Neonatal Network registry of very low birth weight infants with BPD between 2013 and 2017 through retrospective analysis.

**Results:**

A total of 4263 infants were diagnosed with BPD. For mild BPD, infants requiring surgical treatment for patent ductus arteriosus needed a longer LOS [*e*^adjusted β coefficients (adj β)^ 1.041; 95% confidence interval (CI): 0.01–0.08] and hydrocephalus (*e*^adj β^ 1.094; 95% CI 0.01–0.17). In moderate BPD, infants administered steroids or with intraventricular hemorrhage required a longer LOS (*e*^adj β^ 1.041; 95% CI 0.00–0.07 and *e*^adj β^ 1.271; 95% CI 0.11–0.38, respectively). In severe BPD, infants with comorbidities required a longer LOS: pulmonary hypertension (*e*^adj β^ 1.174; 95% CI 0.09–0.23), administrated steroid for BPD (*e*^adj β^ 1.116; 95% CI 0.07–0.14), sepsis (*e*^adj β^ 1.062; 95% CI 0.01–0.11), patent ductus arteriosus requiring surgical ligation (*e*^adj β^ 1.041; 95% CI 0.00–0.08), and intraventricular hemorrhage (*e*^adj β^ 1.016; 95% CI 0.05–0.26). Additionally, the higher the clinical risk index score, the longer the LOS needed for infants in all groups.

**Conclusions:**

The factors affecting LOS differed according to the severity of BPD. Individualized approaches to reducing LOS may be devised using knowledge of the various risk factors affecting LOS by BPD severity.

**Graphical Abstract:**

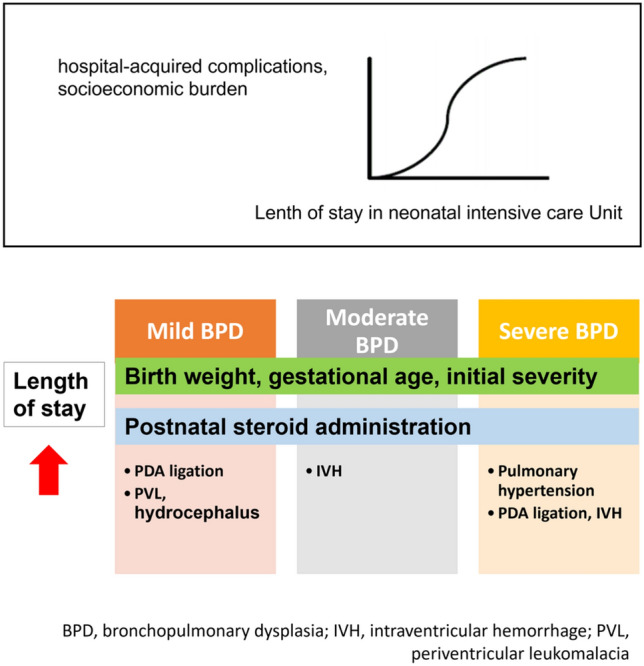

## Introduction

Premature birth, the birth of a baby at less than 37 weeks gestational age (GA), is common and affects 6%–14% of pregnancies [1]. Approximately 15 million premature neonates are born annually worldwide [2]. The survival rate of premature infants with very low birth weight (VLBW) has markedly increased owing to advanced neonatal medicine [3]; however, the improved survival of very preterm infants has increased the incidence of neonatal morbidities in surviving infants.

Bronchopulmonary dysplasia (BPD) was first described by Northway et al. in 1967 [4]. It is one of the most common and serious respiratory sequelae of premature birth [5]. Data from other major cohort studies have demonstrated a BPD prevalence of 11%–56%, depending on GA at birth or birth weight [6–9]. Sufficient fetal lung growth and maturation are key components of postnatal survival [10]. Under normal intrauterine circumstances, the lung begins to develop into a gas exchange organ at 24–28 weeks of gestation; however, lung development in preterm infants is interrupted after birth as they start ventilation, which may require mechanical ventilation and the administration of oxygen. These interruptions in the developing lung may damage premature lungs. BPD results from an aberrant reparative response to both antenatal conditions and repetitive postnatal injuries in the developing lungs of premature infants.

Improvements in survival with BPD have led to more preterm infants requiring longer hospitalization. Infants with severe BPD often spend more than five months [170 days (150–192)] in the neonatal intensive care unit (NICU), with some of the sickest patients spending over a year prior to being discharged home for the first time [11, 12]. Prolonged stays in the NICU are associated with other problems such as delayed establishment of developmental milestones, disturbed familial emotional bonding, increased risk of hospital-acquired nosocomial infections, and substantial socioeconomic burdens on families and societies [13]. Predicting length of stay (LOS) is the cornerstone of medical resource planning. Infants diagnosed with mild, moderate, and severe BPD differ in their baseline characteristics and clinical outcomes [14], therefore it can be expected that the determinants of the length of hospital stay will differ by group. However, a previous study identified a predictor of LOS by analyzing infants regardless of BPD severity [15]. The objective of our study was to identify separately the characteristics associated with longer hospitalization in infants with mild, moderate, or severe BPD. Our findings may help devise an individualized approach based on BPD severity to limit the LOS and reduce the socioeconomic burden from prolonged hospitalizations.

## Methods

The Korean Neonatal Network (KNN), established in 2013 by the Korean Society of Neonatology and the Korea Centers for Disease Control and Prevention, is a nationwide prospective cohort registry of VLBW infants admitted to 77 participating NICUs, covering approximately 70% of the VLBW infants in Korea [16]. The registry includes prospectively collected maternal data and infant outcome data collected from birth until death, transfer, discharge, and after hospital stay using a standardized electronic case report form. Each hospital’s institutional review board (IRB) has approved data collection for the KNN and written informed consent has been obtained from parents at enrollment by every NICU participating in the KNN. All data were monitored regularly by the KNN data management committee and this study was approved by the KNN ethics committee. All methods were performed in accordance with relevant guidelines and regulations.

### Study design and participants

We performed a retrospective cohort study using the KNN registry of VLBW infants born between 24^0/7^ and 31^6/7^ weeks of gestation at participating hospitals from January 1, 2013, to December 31, 2017. Infants diagnosed with BPD were included in this study. Infants who had life-threatening congenital malformations, who died before BPD diagnosis or severity determination, who were admitted to the NICU for > 365 days, who were discharged to general wards or pediatric intensive care units, or who had missing data, were excluded. This study was performed in accordance with the Declaration of Helsinki and was approved by the KNN data management committee and the IRB of Yeouido St. Mary’s Hospital (Approval Number: SC22ZIDI0075).

### Definition of variables

The maternal medical history and postnatal conditions of the included infants were analyzed. Maternal characteristics included age and mode of delivery. Maternal old age is defined as over 35 years at delivery. Maternal hypertension included gestational hypertension, pregnancy-induced hypertension, and chronic hypertension. Maternal diabetes mellitus (DM) included both gestational and overt DM. Histological chorioamnionitis was defined as described by Yoon et al. [17]. Premature and prolonged rupture of membranes (PPROM) was defined as rupture of membranes before delivery if it lasted for > 24 hours. Antenatal corticosteroid (ACS) administration was defined as at least one dose of ACS administered to the mother at any time before delivery to accelerate fetal lung maturity.

Neonatal variables included GA, calculated according to the last menstrual period, birth weight, sex, and length of stay (LOS). The five-minute Apgar score and clinical risk index for babies (CRIB) II score were assessed [18]. Small for gestational age (SGA) was defined as birth weight below the 10th percentile for GA and sex [19]. Respiratory distress syndrome (RDS) was defined as radiological and clinical diagnoses requiring surfactant replacement treatment. Any size of patent ductus arteriosus (PDA) was diagnosed based on echocardiographic and clinical findings. Sepsis was determined based on positive blood culture results. Intraventricular hemorrhage (IVH) was defined as Papile classification grade 3 or 4 on cranial ultrasonography [20]. Cystic periventricular leukomalacia (PVL) was defined as a cyst formation observed at any time in the periventricular white matter on cranial ultrasonography or magnetic resonance imaging of the head. Necrotizing enterocolitis (NEC) was defined as stage II or higher according to Bell’s classification stage [21]. Retinopathy of prematurity (ROP) was defined according to the international classification of ROP [22]; severe ROP was defined as stage 3 or higher, or retinopathy requiring treatment with laser or anti-vascular endothelial growth factor [23].

### Definitions of BPD

BPD was defined as oxygen supplementation for 28 days. The study population was classified into three groups according to the respiratory support status at 36 weeks post-menstrual age based on the definition of BPD adopted by the National Institute of Child Health consensus on BPD severity: mild BPD with no oxygen supplementation, moderate BPD with oxygen supplementation < 30%, and severe BPD with oxygen supplementation ≥ 30% and/or need for positive pressure support [24].

### Statistical analysis

Maternal and neonatal characteristics and neonatal outcomes were analyzed using the chi-square or Kruskal–Wallis test, followed by post hoc Bonferroni’s method and the Dwass–Steel–Critchlow–Fligner method, respectively. The mean LOS according to BPD severity was obtained using a one-way analysis of variance. To identify factors associated with LOS, three linear regression models were conducted separately for the mild, moderate, and severe BPD groups, respectively; variables with *P* < 0.05, as determined by simple linear regression analyses, were considered potential confounders. Variance inflation factors were obtained to examine multicollinearity among the variables. The LOS was skewed, and regression analysis was applied after natural log transformation to normalize the distribution. Both unadjusted and adjusted beta coefficients (adj *β*) with 95% confidence intervals (CI) were reported. Following this, the adj *β* was exponentiated for anti-natural logarithmic transformation. Additionally, post hoc analyses using level 1 as a reference were performed using general linear models in the mild, moderate, and severe BPD groups to compare the effect of CRIB II score levels on mean LOS. All statistical analyses were performed using SAS version 9.4 (SAS Institute, Cary, NC, USA), with two-sided *P *values < 0.05 considered statistically significant.

## Results

### Study populations

**Fig. 1:**
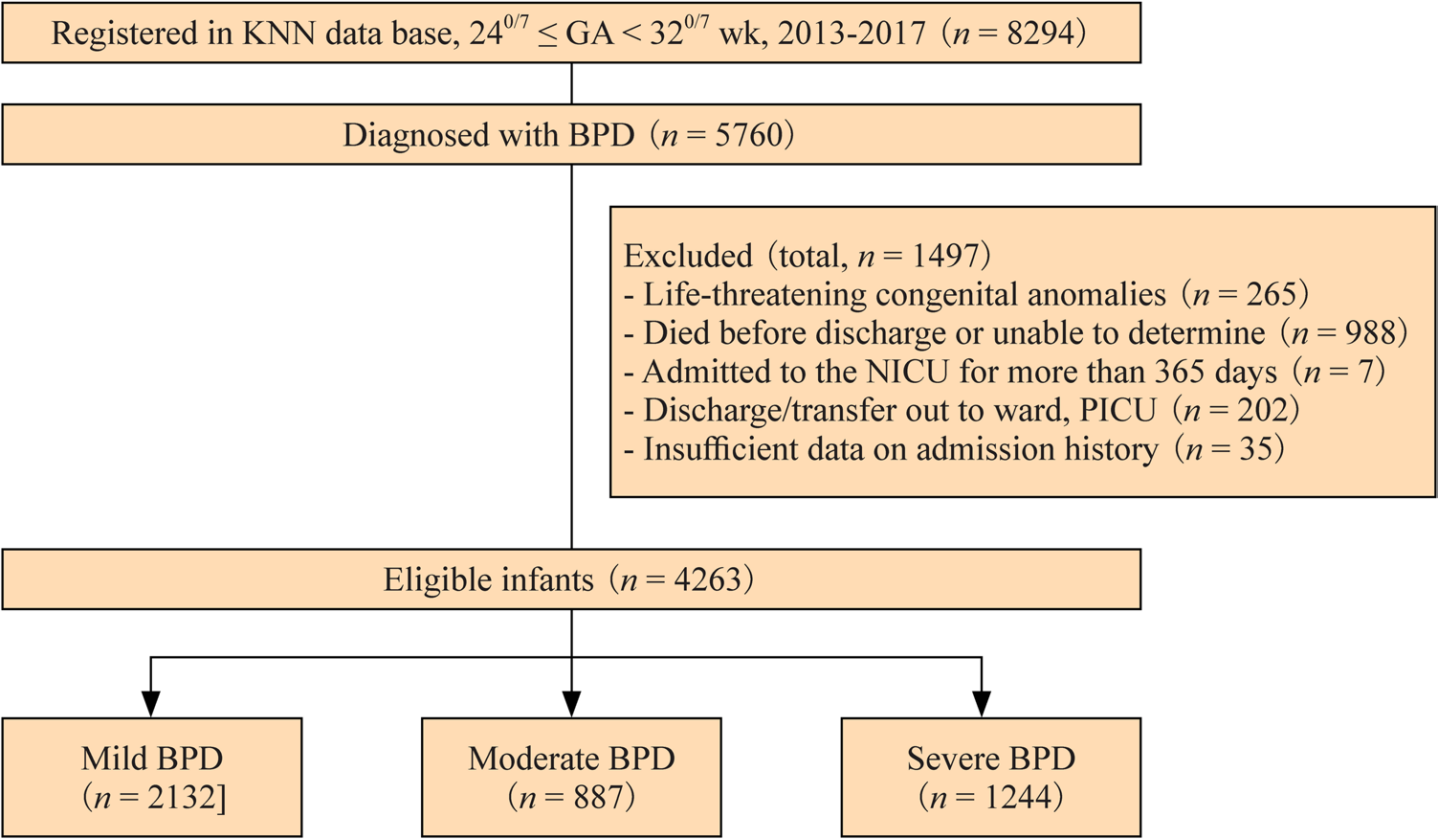
Flow chart of study population. This presents a flowchart of the inclusion and exclusion criteria of our study. Analyzed data [*n* = 4263; mild bronchopulmonary dysplasia (BPD) = 2132, moderate BPD = 887, and severe BPD = 1244] were obtained from the Korean Neonatal Network (KNN) Database, 2013–2017. *GA* gestational age, *NICU* neonatal intensive care unit, *PICU* pediatric intensive care unit

**Fig. 2:**
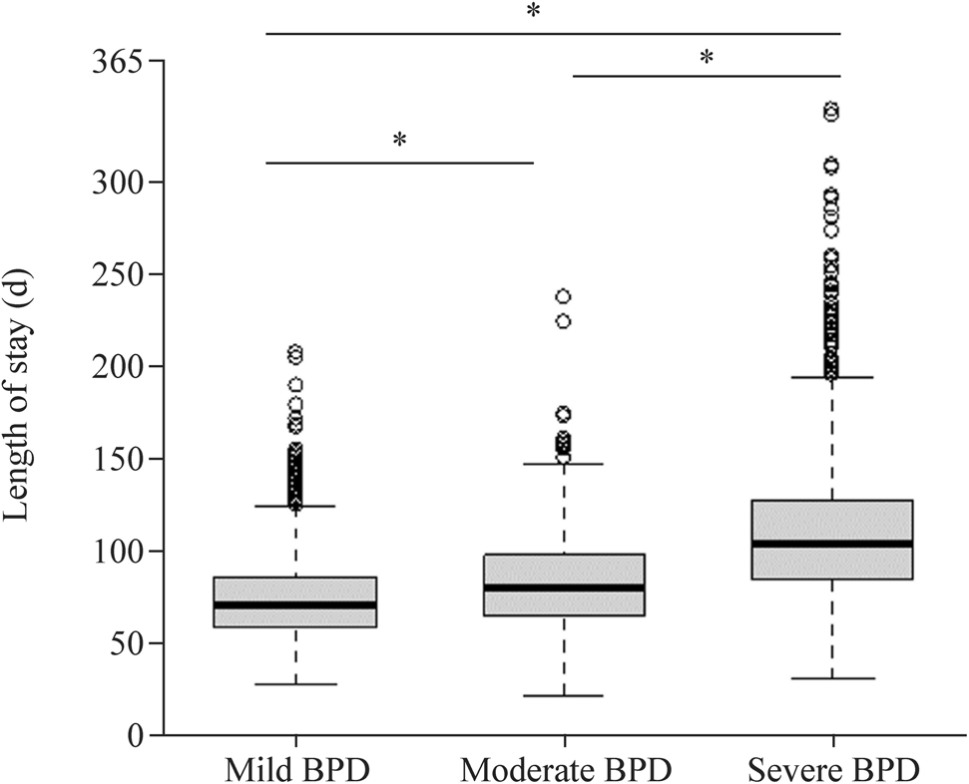
Length of stay according to BPD severity. The length of stay increases with bronchopulmonary dysplasia (BPD) severities: 75.1 ± 21.3 days in the mild BPD group, 84.2 ± 25.5 days in the moderate BPD group, and 111.6 ± 40.3 days in the severe BPD group. ^*^P < 0.001

Of the 8294 VLBW infants with GA between 24^0/7^ and 31^6/7^ weeks born in participating hospitals from January 1, 2013, to December 31, 2017, reviewed as cohorts by accessing the KNN registry, 5760 diagnosed with BPD were selected as the study population. A total of 1492 infants were excluded; 265 infants had life-threatening congenital anomalies, 988 infants died before discharge, seven infants were admitted to the NICU for > 365 days, 202 infants were discharged or transferred to the general ward or pediatric intensive care unit, and 35 infants had insufficient data on either admission history. Finally, 4263 infants were eligible for LOS analyses according to BPD severity: 2132, 887, and 1244 in the mild, moderate, and severe BPD groups, respectively (Fig. 1). The distribution of LOS in each of the three BPD severity groups is shown in Fig. 2 and is as follows: 75.1 ± 21.3 days, 84.2 ± 25.5 days, and 111.6 ± 40.3 days for the mild, moderate, and severe BPD groups, respectively.

### Baseline demographic characteristics

The mean maternal ages of the groups were not significantly different. The proportion of infants with maternal hypertension in the mild BPD group was higher than in the severe BPD group [333 (15.6%) vs. 241 (19.4%); *P* = 0.015]. The percentage of infants whose mothers had oligohydramnios was significantly higher in the severe BPD group than in the other groups [263 (13.6%), 105 (13.6%), and 214 (18.8%) in the mild, moderate, and severe BPD groups, respectively]. The mean birth weight was highest in the mild BPD group and lowest in the severe BPD group, and the mean differences between the three groups were significant. In contrast, the mean GA at birth was lowest in the severe BPD group and highest in the mild BPD group; however, the mean differences were significant only between infants with severe BPD and the others (Table 1).

**Table 1:** Comparison of baseline characteristics in the three BPD groups

Variables	Mild BPD (*n* = 2132)	Moderate BPD (*n* = 887)	Severe BPD (*n* = 1244)	*P*	*Post-hoc* pairwise comparisons		
					Mild vs. Moderate	Mild vs. Severe	Moderate vs. Severe
Maternal characteristics							
Maternal age, y	33.1 ± 4.3	32.9 ± 4.3	33.1 ± 4.2	0.427	0.499	0.969	0.433
Maternal HTN	333 (15.6%)	146 (16.5%)	241 (19.4%)	0.018	> 0.999	0.015	0.258
Maternal DM	212 (9.9%)	94 (10.6%)	102 (8.2%)	0.127	> 0.999	0.276	0.177
Oligohydramnios	263 (13.6%)	105 (13.6%)	214 (18.8%)	< 0.001	> 0.999	< 0.001	0.006
ACS	1760 (83.5%)	704 (81.4%)	1035 (84.6%)	0.145	0.525	> 0.999	0.153
HCAM	698 (38.6%)	312 (40.1%)	453 (43.3%)	0.048	> 0.999	0.042	0.489
PROM	830 (39.2%)	366 (41.7%)	481 (39.0%)	0.377	0.597	> 0.999	0.642
CS	1613 (75.7%)	661 (74.5%)	982 (78.9%)	0.033	> 0.999	0.087	0.051
Multiplets	736 (34.5%)	284 (32.0%)	375 (30.1%)	0.029	0.555	0.027	> 0.999
Infantile characteristics							
BW, g	1059.7 ± 222.6	1033.4 ± 239.6	921.4 ± 242.9	< 0.001	0.006	< 0.001	< 0.001
GA, wk	27.9 ± 1.6	27.8 ± 1.9	27.3 ± 2.0	< 0.001	0.738	< 0.001	< 0.001
Male gender	1063 (49.9%)	495 (55.8%)	691 (55.5%)	0.001	0.009	0.003	> 0.999
5’ AS	6.8 ± 1.6	6.3 ± 1.7	6.3 ± 1.8	< 0.001	< 0.001	< 0.001	0.908
CRIB II score	8.5 ± 2.7	8.8 ± 2.9	9.8 ± 3.1	< 0.001	< 0.001	< 0.001	< 0.001
Level 1 (≤ 5)	231 (14.4%)	95 (14.5%)	88 (9.8%)				
Level 2 (> 5– ≤ 10)	1048 (65.4%)	363 (55.5%)	426 (47.3%)				
Level 3 (> 10– ≤ 15)	312 (19.5%)	192 (29.4%)	369 (41.0%)				
Level 4 (> 15)	12 (0.7%)	4 (0.6%)	17 (1.9%)				
SGA	74 (6.9%)	27 (6.4%)	110 (17.6%)	< 0.001	> 0.999	< 0.001	< 0.001

Values are expressed as mean ± standard deviation or frequencies (%)

*P* values were calculated using the chi-square test with post hoc Bonferroni method or Kruskal–Wallis test with post hoc DwassSteel-Critchlow Fligner method

*ACS* antenatal corticosteroid, *BW* birth weight, *BPD* bronchopulmonary dysplasia, *CS* Cesarean section, *GA* gestational age, *DM* diabetes mellitus, *HCAM* histological chorioamnionitis, *HTN* hypertension, *PROM* premature rupture of membrane, *5’ AS* 5 min Apgar score, *CRIB* clinical risk index for babies, *SGA* small for gestational age

### Neonatal outcomes

The neonatal outcomes differed depending on the severity of BPD (Table 2). Most infants were diagnosed with RDS, and the proportion was highest in the severe BPD group [1976 (92.7%) in the mild BPD group, 823 (92.8%) in the moderate BPD group, and 1184 (95.2%) in the severe BPD group]. However, the differences were significant only between the mild and severe BPD groups (*P* = 0.012). Fifty-three infants (2.5%) with mild BPD, 41 infants (4.6%) with moderate BPD, and 82 (6.6%) infants developed air leaks, and the differences between mild BPD and other groups were significant (*P* = 0.006 for moderate BPD and *P* < 0.001 for severe BPD group). The incidence of massive pulmonary hemorrhage increased with BPD severity to 70 (3.3%), 41 (4.6%), and 103 (8.3%) in the mild, moderate, and severe BPD groups, respectively. The differences between the severe and mild BPD groups (*P* < 0.001) and between the severe and moderate BPD groups (*P* = 0.003) were significant. Infants with pulmonary hypertension, sepsis, PDA requiring surgical treatment, or IVH who were administered postnatal steroids also showed statistically significant increases in the severity of BPD. As BPD severity increased, more infants were diagnosed with PVL and posthemorrhagic hydrocephalus (PHH); however, differences were not significant between the moderate and severe BPD groups. The proportion of neonatal outcomes such as NEC or severe ROP also increased with BPD severity; however, the differences were not significant between the mild and moderate BPD groups.

**Table 2:** Comparison of neonatal outcomes in three groups

Variables	Mild BPD (*n* = 2132)	Moderate BPD (*n* = 887)	Severe BPD (*n* = 1244)	*P*	*Post-hoc* pairwise comparisons		
					Mild vs. moderate	Mild vs. severe	Moderate vs. severe
RDS	1976 (92.7%)	823 (92.8%)	1184 (95.2%)	0.013	> 0.999	0.012	0.060
Air leak	53 (2.5%)	41 (4.6%)	82 (6.6%)	< 0.001	0.006	< 0.001	0.165
MPH	70 (3.3%)	41 (4.6%)	103 (8.3%)	< 0.001	0.225	< 0.001	0.003
Pulmonary hypertension	51 (2.4%)	50 (5.6%)	194 (15.6%)	< 0.001	< 0.001	< 0.001	< 0.001
Postnatal steroid	533 (25.0%)	387 (43.6%)	771 (62.0%)	< 0.001	< 0.001	< 0.001	< 0.001
Sepsis	90 (4.2%)	76 (8.6%)	175 (14.1%)	< 0.001	< 0.001	< 0.001	< 0.001
Surgical PDA	219 (13.9%)	146 (20.9%)	343 (34.6%)	< 0.001	< 0.001	< 0.001	< 0.001
NEC	104 (4.9%)	52 (5.9%)	135 (10.9%)	< 0.001	0.798	< 0.001	< 0.001
Severe ROP	187 (8.8%)	101 (11.4%)	302 (24.3%)	< 0.001	0.075	< 0.001	< 0.001
IVH	118 (5.5%)	79 (8.9%)	163 (13.1%)	< 0.001	0.003	< 0.001	0.009
PVL	150 (7.0%)	96 (10.8%)	164 (13.2%)	< 0.001	0.003	< 0.001	0.300
PHH	53 (2.5%)	43 (4.9%)	85 (6.8%)	< 0.001	0.003	< 0.001	0.171
LOS, d	75.1 ± 21.3	84.2 ± 25.5	111.6 ± 40.3	< 0.001	< 0.001	< 0.001	< 0.001

Values are expressed as mean ± standard deviation or frequencies (%)

*P* values were calculated using the chi-square test with post hoc Bonferroni method or Kruskal–Wallis test with post hoc DwassSteel-Critchlow Fligner method

*BPD* bronchopulmonary dysplasia, *IVH* intraventricular hemorrhage, *MPH* massive pulmonary hemorrhage, *LOS* length of stay, *NEC* necrotizing enterocolitis, *PDA* patent ductus arteriosus, *PHH* post hemorrhagic hydrocephalus, *PVL* periventricular leukomalacia, *RDS* respiratory distress syndrome, *ROP* retinopathy of prematurity

### Factors affecting length of stay

As the LOS was skewed, linear regression models were used after natural log transformation. In the multivariate regression models for LOS, maternal factors, infantile factors, and combined neonatal morbidities (outcome) had differential effects according to the BPD severity strata. Maternal factors were not associated with LOS except for maternal hypertension in the severe BPD group. In the multivariable analyses, the linear regression model showed that in the severe BPD group, stays for infants with maternal hypertension were 0.932 times shorter than that for infants without maternal hypertension [exponentiate adj β (*e*^adj β^) 0.932; 95% CI  −  0.11 to  − 0.02; *P* = 0.006].

Multivariable analyses confirmed that LOS was longer in cases of extremely low birth weight (ELBW, birth weight < 1000 g) or very preterm birth (GA < 28 weeks) in all three BPD groups. ELBW infants had longer stays in the NICU (*e*^adj β^ 1.127, 95% CI 0.10–0.15; *e*^adj β^ 1.185, 95% CI 0.13–0.20; and *e*^adj β^ 1.139, 95% CI 0.09–0.18, for mild, moderate, and severe BPD, respectively). Very preterm infants stayed longer in the NICU (*e*^adj β^ 1.174, 95% CI 0.14–0.19; *e*^adj β^ 1.197; 95% CI 0.14–0.22; and *e*^adj β^ 1.127; 95% CI 0.07–0.16, for mild, moderate, and severe BPD, respectively). High-risk infants at admission, as evaluated by the CRIB II score, stayed longer in the NICU. Interestingly, the higher the score, the longer the LOS in all severity groups, increasing adj β. If infants had a CRIB II score > 15 at admission, adj β varied 0.34, 0.38, and 0.28 for mild, moderate, and severe BPD respectively (*e*^adj β^ 1.405, 95% CI 0.22–0.47; *e*^adj β^ 1.462, 95% CI 0.15–0.61; and *e*^adj β^ 1.323, 95% CI 0.12–0.44, for mild, moderate, and severe BPD, respectively).

In the mild BPD group, infants requiring surgical treatment for PDA, diagnosed with PVL or PHH needed an increased LOS (*e*^adj β^ 1.041, 95% CI 0.01–0.08; *e*^adj β^ 1.094, 95% CI 0.03–0.15; and *e*^adj β^ 1.094, 95% CI 0.01–0.17, respectively). In the moderate BPD group, infants who were administered steroids or diagnosed with IVH required a longer LOS (*e*^adj β^ 1.041, 95% CI 0.00–0.07 and *e*^adj β^ 1.271, 95% CI 0.11–0.38, respectively). In the severe BPD group, most pulmonary outcomes were associated with LOS (*e*^adj β^ 1.174, 95% CI 0.09–0.23 for pulmonary hypertension; *e*^adj β^ 1.116, 95% CI 0.07–0.14 for postnatal steroids). If infants were diagnosed with sepsis or PDA requiring surgical treatment, they were discharged later from the NICU (*e*^adj β^ 1.062, 95% CI 0.01–0.11 and *e*^adj β^ 1.041, 95% CI 0.00–0.08, respectively). Infants who developed IVH needed more days in the NICU (*e*^adj β^ 1.016, 95% CI 0.05–0.26) (Table 3).

**Table 3:** Linear regression model of factors associated with an increased length of stay three groups

Variables	Mild BPD (*n* = 2132)			Moderate BPD (*n* = 887)			Severe BPD (*n* = 1244)		
	*β* (95% CI)	adj *β* (95% CI)	*e*^adj* β*^	*β* (95% CI)	adj *β* (95% CI)	*e*^adj* β*^	*β* (95% CI)	adj *β* (95% CI)	*e*^adj* β*^
Maternal characteristics									
Maternal old age	0.00 (− 0.03, 0.03)			− 0.01 (− 0.06, 0.04)			0.02 (− 0.02, 0.06)		
Maternal HTN	− 0.03 (− 0.06, 0.00)			− 0.05 (− 0.11, 0.00)			** − 0.10 (− 0.15, − 0.05)**^**‡**^	** − 0.07 (− 0.11, − 0.02)**^**†**^	**0.932**
Maternal DM	** − 0.04 (− 0.08, 0.00)**	− 0.02 (− 0.05, 0.01)		− 0.10 (− 0.16, − 0.03)	− 0.03 (− 0.08, 0.02)		− 0.07 (− 0.14, 0.00)		
Oligohydramnios	0.01 (− 0.03, 0.04)			0.00 (− 0.06, 0.07)			0.05 (− 0.01, 0.10)		
ACS	**0.04 (0.01, 0.07)**	0.03 (− 0.01, 0.06)		− 0.01 (− 0.06, 0.04)			0.01 (− 0.04, 0.07)		
HCAM	**0.05 (0.03, 0.08)**^**‡**^	0.01 (− 0.01, 0.03)		0.10 (0.06, 0.14)^‡^	0.03 (− 0.00, 0.06)		0.05 (0.01, 0.09)^*^	− 0.01 (− 0.05, 0.02)	
PROM	0.00 (− 0.02, 0.03)			0.01 (− 0.03, 0.05)			0.03 (− 0.01, 0.07)		
CS	− 0.02 (− 0.04, 0.01)			− 0.05 (− 0.10, 0.00)	− 0.01 (− 0.05, 0.03)		− 0.04 (− 0.09, 0.01)		
Multiplets	− 0.02 (− 0.04, 0.01)			− 0.04 (− 0.08, 0.00)			− 0.01 (− 0.06, 0.03)		
Infantile characteristics									
BW < 1000 g	**0.29 (0.27, 0.31)**^**‡**^	**0.12 (0.10, 0.15)**^**‡**^	**1.127**	**0.32 (0.29, 0.36)**^**‡**^	**0.17 (0.13, 0.20)**^**‡**^	**1.185**	**0.30 (0.26, 0.33)**^**‡**^	**0.13 (0.09, 0.18)**^**‡**^	**1.139**
GA < 28 wk	**0.31 (0.29, 0.33)**^**‡**^	**0.16 (0.14, 0.19)**^**‡**^	**1.174**	**0.34 (0.31, 0.37)**^**‡**^	**0.18 (0.14, 0.22)**^**‡**^	**1.197**	**0.32 (0.28, 0.35)**^**‡**^	**0.12 (0.07, 0.16)**^**‡**^	**1.127**
Male	− 0.01 (− 0.03, 0.01)			− 0.01 (− 0.05, 0.03)			− 0.01 (− 0.05, 0.03)		
5’ AS < 7	**0.06 (0.04, 0.08)**^**‡**^	0.01 (− 0.01, 0.03)		**0.11 (0.07, 0.16)**^**‡**^	0.03 (− 0.00, 0.07)		**0.11 (0.07, 0.14)**^**‡**^	− 0.01 (− 0.05, 0.03)	
CRIB II score^a^									
Level 1 (≤ 5)		Reference			Reference			Reference	
Level 2 (> 5– ≤ 10)	**0.24 (0.21, 0.27)**^**‡**^	**0.11 (0.08, 0.14)**^**‡**^	**1.116**	**0.20 (0.21, 0.31)**^**‡**^	**0.11 (0.05, 0.16)**^**‡**^	**1.116**	**0.29 (0.22, 0.36)**^**‡**^	**0.14 (0.06, 0.22)**^**‡**^	**1.150**
Level 3 (> 10– ≤ 15)	**0.49 (0.46, 0.53)**^**‡**^	**0.18 (0.14, 0.22)**^**‡**^	**1.197**	**0.55 (0.49, 0.61)**^**‡**^	**0.17 (0.10, 0.24)**^**‡**^	**1.185**	**0.54 (0.47, 0.61)**^**‡**^	**0.24 (0.15, 0.33)**^**‡**^	**1.271**
Level 4 (> 15)	**0.69 (0.56, 0.82)**^**‡**^	**0.34 (0.22, 0.47)**^**‡**^	**1.405**	**0.77 (0.54, 1.00)**^**‡**^	**0.38 (0.15, 0.61)**^**†**^	**1.462**	**0.67 (0.52, 0.82)**^**‡**^	**0.28 (0.12, 0.44)**^**†**^	**1.323**
SGA	**0.10 (0.04, 0.16)**^**‡**^	0.05 (0.00, 0.10)		0.04 (− 0.08, 0.15)			0.05 (− 0.03, 0.12)		
Neonatal outcome									
RDS	**0.14 (0.10, 0.19)**^**‡**^			**0.24 (0.17, 0.32)**^**‡**^			**0.14 (0.05, 0.23)**^**‡**^		
Air leak	**0.10 (0.02, 0.17)**^**†**^	0.01 (− 0.05, 0.07)		0.10 (0.00, 0.19)^*^	0.00 (− 0.07, 0.08)		**0.11 (0.03, 0.19)**^**†**^	0.04 (0.02, 0.11)	
MPH	**0.08 (0.02, 0.15)**^**†**^	− 0.01 (− 0.07, 0.04)		0.08 (− 0.01, 0.18)			0.08 (0.01, 0.15)^*^	− 0.03 (− 0.10, 0.03)	
Pulmonary hypertension	**0.10 (0.02, 0.17)**^**†**^	− 0.09 (− 0.19, 0.00)		**0.13 (0.04, 0.22)**^**†**^	− 0.06 (− 0.17, 0.05)		**0.25 (0.20, 0.30)**^**‡**^	**0.16 (0.09, 0.23)**^**‡**^	**1.174**
Postnatal steroid	**0.18 (0.15, 0.20)**^**‡**^	**0.07 (0.05, 0.09)**^**‡**^	**1.073**	**0.13 (0.09, 0.17)**^**‡**^	0.04 (0.00, 0.07)^*^	**1.041**	**0.20 (0.16, 0.23)**^**‡**^	**0.11 (0.07, 0.14)**^**‡**^	**1.116**
Sepsis	**0.16 (0.10, 0.22)**^**‡**^	0.03 (− 0.02, 0.09)		0.09 (0.02, 0.17)^*^	0.03 (− 0.03, 0.09)		**0.18 (0.13, 0.24)**^**‡**^	0.06 (0.01, 0.11)^*^	**1.062**
Surgical PDA	**0.14 (0.11, 0.18)**^**‡**^	**0.04 (0.01, 0.08)**^**†**^	**1.041**	**0.15 (0.09, 0.20)**^**‡**^	0.03 (− 0.01, 0.08)		**0.13 (0.08, 0.17)**^**‡**^	0.04 (0.00, 0.08)^*^	**1.041**
NEC	**0.15 (0.10, 0.20)**^**‡**^	0.01 (− 0.04, 0.07)		0.09 (0.02, 0.16)^*^	− 0.01 (− 0.09, 0.07)		**0.16 (0.11, 0.22)**^**‡**^	0.04 (− 0.04, 0.11)	
Severe ROP	**0.05 (0.01, 0.10)**^**†**^	0.03 (− 0.01, 0.07)		− 0.02 (− 0.08, 0.05)			0.07 (0.02, 0.13)^*^	0.02 (− 0.04, 0.07)	
PVL	**0.23 (0.18, 0.28)**^**‡**^	**0.09 (0.03, 0.15)**^**†**^	**1.094**	**0.16 (0.08, 0.25)**^**‡**^	− 0.03 (− 0.11, 0.05)		**0.13 (0.07, 0.19)**^**‡**^	− 0.02 (− 0.11, 0.06)	
IVH	**0.31 (0.27, 0.35)**^**‡**^	0.09 (0.00, 0.17)		**0.31 (0.25, 0.37)**^**‡**^	0.24 (0.11, 0.38)^*^	**1.271**	**0.24 (0.20, 0.29)**^**‡**^	**0.16 (0.05, 0.26)**^**†**^	**1.016**
PHH	**0.22 (0.14, 0.29)**^**‡**^	0.09 (0.01, 0.17)^*^	**1.094**	**0.13 (0.04, 0.22)**^**†**^	0.03 (− 0.08, 0.14)		**0.16 (0.09, 0.24)**^**†**^	0.06 (− 0.03, 0.15)	

The LOS was skewed; therefore, simple and multiple linear regression models were used after natural log transformation of the data. Multiple linear regression analyses were performed, adjusted for variables with a *P* < 0.05 in simple linear regression analysis, except for RDS (due to multicollinearity), to assess the associations with LOS. After completion of analyses, we inversed natural log-transformed adjusted *β*, exponential (adjusted *β*)

^*^P < 0.05, ^†^*P* < 0.01, ^‡^*P* < 0.001

^a^ The CRIB II score was further classified into four levels: level 1:0 to 5, level 2:6 to 10, level 3:11 to 15, and level 4 above 15. There were no infants with level 1. Adjusted *β* were calculated by CRIB II score level 1, as reference

Bold values indicate significant *P* values

*ACS* antenatal corticosteroid, *AS* Apgar score, *BW* birth weight, *CS* Cesarean section, *DM* diabetes mellitus, *CRIB* clinical risk index for babies, *GA* gestational age, *HCAM* histological chorioamnionitis, *HTN* Hypertension, *IVH* intraventricular hemorrhage, *MPH* massive pulmonary hemorrhage, *NEC* necrotizing enterocolitis, *PDA* patent ductus arteriosus, *PHH* post hemorrhagic hydrocephalus, *PROM* premature rupture of membrane, *PVL* periventricular leukomalacia, *RDS* respiratory distress syndrome, *ROP* retinopathy of prematurity, *SGA* small for gestational age

## Discussion

We found that the risk factors associated with longer LOS differed for infants, depending on BPD severity (mild, moderate, or severe). We believe that this study makes a significant contribution to the literature because more individualized approaches to reducing LOS could be possible with this information. There were several notable findings in our study, including differences between the BPD groups. First, we found that the initial conditions of infants, such as GA at birth, birth weight, and CRIB II score, were independently associated with LOS, regardless of BPD severity. Second, maternal factors had little effect on the LOS, except for maternal hypertension in infants with severe BPD. Lastly, infants in the mild BPD group requiring surgical treatment during hospitalization in the NICU were independently associated with LOS, but the accompanying comorbidities were more important in infants in the severe BPD group.

### Differences between groups

Several studies have found that the incidence of BPD increases as the GA at birth and birth weight decrease [25]. Unlike previous studies, we divided the study population into three groups according to the severity of BPD and investigated them separately. We found that the severity of BPD increased, as GA at birth and birth weight of the infant decreased. Lower birth weight and premature birth were associated with an increased risk of developing severe BPD, consistent with a previous report by Han et al. [14]. Interestingly the CRIB II scores differed by group. As the CRIB II score assesses an infant’s initial illness at admission, the initial condition of the infant influences the severity of BPD.

In addition to the immature lungs of preterm infants, exposure to maternal oligohydramnios in the intrauterine period adversely affects fetal lung development and easily becomes dysplastic [26]. The highest rate of maternal oligohydramnios in the severe BPD group in our study is consistent with that reported in previous studies [27, 28] (Table 1).

### Common factors affecting length of stay

There was little difference in the basic demographic information between the groups based on BPD severity. GA at birth and birth weight significantly impacted LOS, regardless of BPD severity. Using an anti-logarithmic conversion (*e*^adj β^), we could identify the factors common to all three BPD severity groups. The LOS increased in ELBW infants by 1.1–1.2 times compared to non-ELBW infants. The LOS of very preterm infants < 28 weeks of gestation at birth was also 1.1–1.2 times longer than that of infants ≥ 28 weeks of gestation at birth. This result conforms with a recent systematic review not limited to infants with BPD; GA and/or birth weight have also been identified as critical risk factors affecting the initial LOS [29]. The earlier the GA at birth, the more days are expected for maturation until the term-equivalent age; therefore, the LOS will become longer. Immaturity, represented by GA at birth, is a very strong factor and is known to be an important risk factor for major neonatal morbidities [30]. As LOS can be considered to reflect neonatal morbidities, we confirmed that it was affected by GA at birth and birth weight in all groups regardless of the severity of BPD.

Infants who were administered postnatal steroids had longer LOS than those not treated with steroids: the percent increase in LOS was 7.3%, 4.1%, and 11.6% for the mild, moderate, and severe BPD groups, respectively. Postnatal steroids can be administered to infants at various times, but administration of steroids soon after birth advantageously relieves the inflammatory cascade that leads to BPD development. Conversely, delaying treatment and using more selective criteria, such as the continued need for ventilatory support, can help identify infants most likely to develop BPD [31]. In addition, infants with more severe symptoms and signs who are diagnosed with severe BPD are less likely to benefit from steroid treatment and cannot be discharged earlier due to the continued need for ventilation [32]. This may explain the markedly prolonged LOS observed in the severe BPD group with regard to postnatal steroids.

### Different BPD severity groups

Surgically treated PDA was independently associated with prolonged LOS in the mild BPD group. Similar results were recently published in South Korea; infants who underwent PDA ligation stayed longer in the NICU than those who did not [33]. The optimal timing for surgical ligation of PDA remains controversial. According to a recently published meta-analysis, early surgical ligation of PDA might have a better respiratory outcome and nutritional status than late surgical ligation [34]. Usually, surgical ligation is considered after medical treatment for PDA has failed or is contraindicated. Therefore, the timing of ligation is relatively later than that of medical treatment, and infants who require this surgery are treated for longer and have a longer LOS [35]. Infants in the mild BPD group were born at an older gestational age and needed fewer days to reach the term-equivalent age to be discharged than the others, and the timing of surgical ligation for PDA could be close to the term-equivalent age. In other words, surgically treated PDA might be a determinant of the prolonged LOS in the mild BPD group. Meanwhile, the requirement for surgical treatment for PDA was not a significant risk factor for increased LOS in infants in the moderate BPD group. As surgical ligation of the PDA is performed within two weeks in most cases [36], which is too early to discharge from the NICU for very preterm infants, born before 32 weeks of gestation. This factor increases the LOS in the NICU of infants with mild BPD. A few weeks after severe IVH, PHH may develop because of disturbances in Cerebrospinal fluid flow and absorption [37]. Additionally, white matter damage secondary to PHH is likely to be exacerbated by compression and ischemia due to increased intracranial pressure [38]. As preterm infants grow, their condition worsens or symptoms develop, and treatment is required, resulting in longer hospitalization [39].

Unlike the mild BPD group, the LOS of infants in the severe BPD group was affected by several neonatal outcomes, such as pulmonary hypertension, IVH, and sepsis. Higher incidences of neonatal outcomes in the severe BPD group were also found compared with those of mild and moderate BPD groups, results that are in line with a previous study [40]. These findings could reflect that infants in the severe BPD group had been affected by various comorbidities, easily. Although it was unclear whether combined comorbidities were directly associated with longer LOS, Hintz et al. reported the relationship between clinical comorbidity and longer LOS in NICU [41]. Therefore, combined comorbidities in the severe BPD group might have associations with prolonged LOS in NICU.

### Initial illness severity

The risk level for preterm neonates can be determined based on their initial clinical condition owing to the immaturity of their structural and functional organs. Scoring systems developed in neonatal medicine for estimating mortality and morbidities can be applied to LOS predictions by accounting for the severity of illness in the first week of life beyond factors known at birth [42]. From our regression model, the CRIB II score can also help predict LOS even after adjusting for several confounding factors. Moreover, there were interesting dose–response relationships between the CRIB II score and LOS. Infants with severe initial illness were observed to require a longer LOS. Infants with a level 2 or 3 CRIB II score had a 1.1–1.2 times longer LOS than level 1 infants, while the most severely affected CRIB II level 4 infants had a 1.4–1.5 times longer LOS. The worse the initially evaluated CRIB II score, the longer the hospitalization period, which was observed in mild, moderate, and severe BPD groups. Considering these dose–response relationships, different cut-off points of the CRIB II score can predict longer hospitalization in each BPD group. Advanced neonatal care by skilled personnel is important to shorten hospitalization from resuscitation immediately after delivery to initial management after NICU admission.

The strengths of our study were that we used data from a population-based national cohort covering about 70% of VLBW infants in Korea and that the KNN uses a meticulous data collection system, without biases arising from differences between hospitals or individual neonatal units due to local discharge practices within units [16]. The KNN maintains a complete data-monitoring system to improve data quality [43]. However, this study has several limitations. Because the LOS values were not normally distributed, we log-transformed them and interpreted the results after the anti-logarithmic transformation of adj *β*. The second limitation was the use of registry data. Actual practices such as ventilator weaning protocol may inevitably differ across participating centers, and information about the exact size or hemodynamic significance of PDA could not be obtained. Third, longer LOS may have been underestimated. Infants transferred to the pediatric intensive care unit or general ward were excluded because the actual discharge date from the hospital was indistinguishable. Finally, along with reducing the socioeconomic burden, the overarching goal of reducing BPD severity for preterm infants should be considered to improve long-term neurodevelopmental outcomes for this cohort.

In conclusion, our study identified critical risk factors associated with extended initial hospitalization in preterm infants with mild, moderate, and severe BPD, respectively. Risk factors for longer LOS varied across different levels of severity of BPD. A unique approach is needed to reduce the LOS for infants in each BPD group, and this might be an important suggestion considering the socioeconomic burden. In addition, these risk factors may serve to predict the duration of initial hospitalization, which would be valuable to parents and families, clinicians, and other service providers.

In the future, shortening LOS could be possible by individualized approaches according to the BPD severity of the infants. For example, setting of management protocol through quality improvement (QI) programs including better neonatal resuscitation at birth, efforts to minimize accompanying comorbidities, and specific protocols for postnatal steroid use facilitates reducing LOS. These efforts, directed at reducing the LOS connected to the reduction of preterm morbidities including BPD, are ultimately aimed at having a better neurodevelopmental outcome.

## Data Availability

The data supporting the findings of this study are available from the Korean Neonatal Network (KNN). The information contained in the data must be protected as confidential and will only become available to those individuals who have obtained permission from KNN to access and use these data for a permitted research activity.
